# Long-term follow-up of voice changes after cervical mediastinoscopy

**DOI:** 10.1186/s13019-022-01884-w

**Published:** 2022-06-18

**Authors:** Ikram Achbar, Wilson W. L. Li, Simone T. Timman, Stefan M. van der Heide, Olga C. J. Schuurbiers, Erik H. F. M. van der Heijden, Ad F. T. M. Verhagen

**Affiliations:** 1 Department of Cardiothoracic Surgery, Radboud University Medical Centre, P.O. Box 9101, 6500 HB Nijmegen, The Netherlands; 2 Department of Pulmonary Diseases, Radboud University Medical Centre, P.O. Box 9101, 6500 HB Nijmegen, The Netherlands

**Keywords:** Cervical mediastinoscopy; Vocal cord palsy; Recurrent laryngeal nerve

## Abstract

**Background:**

Vocal cord palsy after cervical mediastinoscopy is usually reported at less than 1%. However, its incidence might be underestimated and no follow-up studies are available. Our study aimed to evaluate the incidence of voice changes after cervical mediastinoscopy and report on long-term outcomes, including quality of life, after at least one-year follow-up.

**Methods:**

A retrospective cohort study was performed, considering all patients who underwent cervical mediastinoscopy in our center between January 2011 and April 2016. Patients with pre-existing voice changes, voice changes only after pulmonary resection and patients who underwent neoadjuvant chemo(radio)therapy were excluded. Voice changes with full recovery within 14 days were attributed to intubation-related causes. Follow-up questionnaires, including the standardized Voice Handicap Index, were sent to patients with documented voice changes.

**Results:**

Of 270 patients who were included for final analysis, 17 (6.3%) experienced voice changes after cervical mediastinoscopy, which persisted > 2 years in 4 patients (1.5%), causing mild to moderate disabilities in daily living. Twelve patients (out of 17, 71%) were referred for otolaryngology consultation, and paresis of the left vocal cord suggesting recurrent laryngeal nerve injury was confirmed in 10 (3.7% of our total study group). Additionally, 83% of the patients who were referred for otolaryngology consultation received voice treatment. Recovery rate after vocal exercises therapy and injection laryngoplasty was respectively 71% and 33%.

**Conclusions:**

Voice changes after cervical mediastinoscopy is an underreported complication, with an incidence of at least 6.3% in our retrospective study, with persisting complaints in at least 1.5% of patients, leading to mild to moderate disabilities in daily living. These findings highlight the need for appropriate patient education for this underestimated complication, as well as the exploration of possible preventive measures.

## Background

Vocal cord palsy due to recurrent laryngeal nerve (RLN) injury is a well-known, albeit infrequently reported complication after cervical mediastinoscopy (CM) [1, 2], usually presenting as voice changes such as dysphonia or hoarseness postoperatively. In most case series, it is reported at less than 1% [2, 3]. However, its incidence might be underestimated. When routine indirect laryngoscopy was performed after CM in a historical series [4], vocal cord palsy was detected in 6% of patients, suggesting this problem might be overlooked during regular clinical follow-up. Additionally, when the extensiveness of mediastinal lymph node dissection is increased, i.e. with transcervical extended mediastinal lymphadenectomy (TEMLA) or video-assisted mediastinoscopic lymphadenectomy (VAMLA) techniques, the risk of RLN injury also rises [4]. In a contemporary series with 108 patients after VAMLA, recurrent nerve palsy was identified in 5% of patients [5]. It has been demonstrated that RLN injury is predominantly caused by traction intraoperatively, mostly due to digital dissection [1]. Therefore, it can be assumed this is a transient problem. However, no follow-up studies are available to verify this assumption, and no data is available to evaluate recovery rate, or the effect this complication has on patients’ QoL.

In the current study, we evaluate the incidence of voice changes after CM, and report on the outcomes of these patients after at least one-year follow-up, with specific emphasis on the impact of voice impairment on QoL.

## Methods

**Fig. 1:**
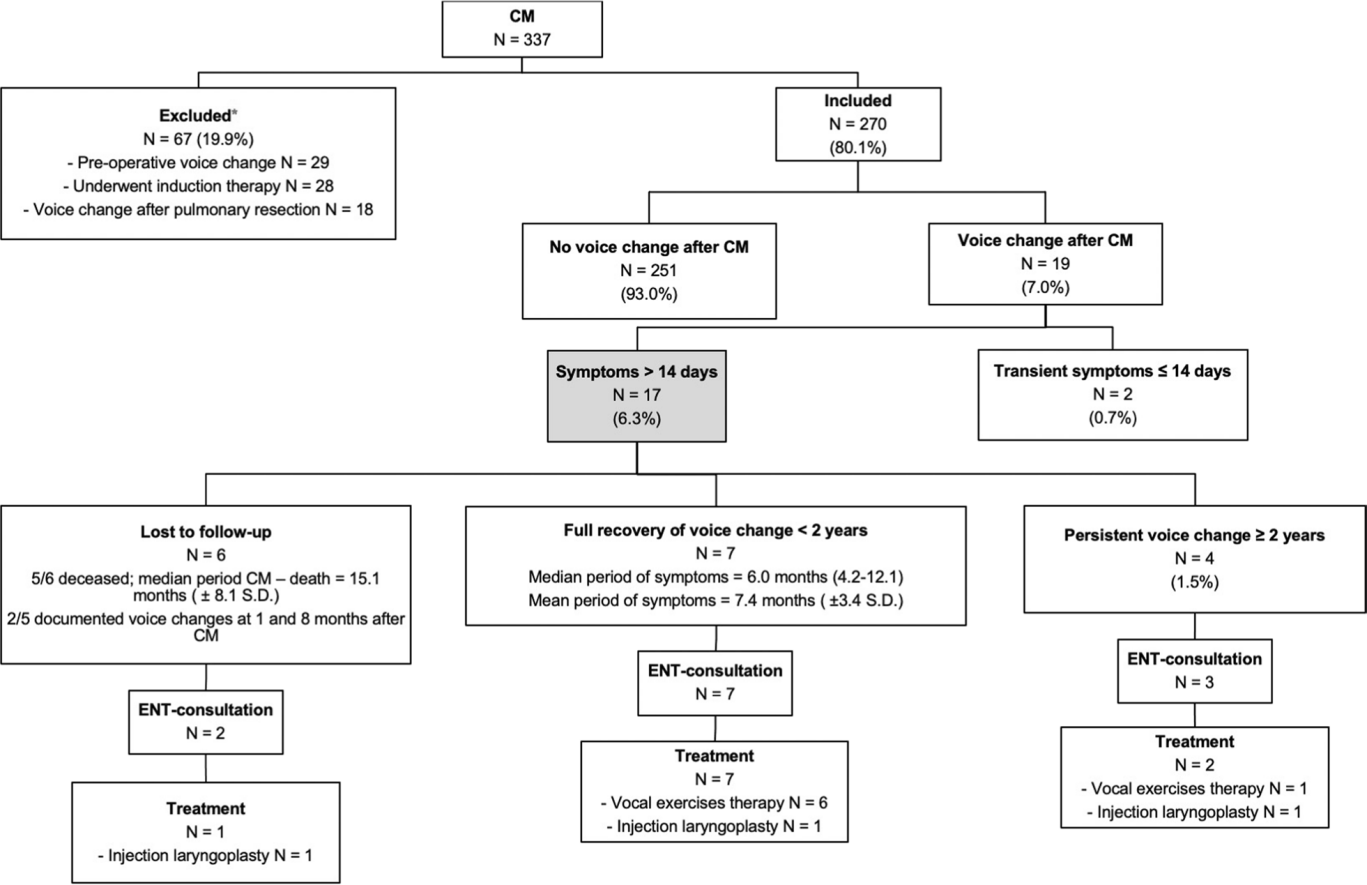
Study patient flow diagram. *Patients can be excluded because of multiple reasons. CM = Cervical Mediastinoscopy. S.D. = Standard Deviation. ENT: Ear Nose Throat/Otolaryngology

We performed a single-center retrospective cohort study, for which ethical approval was waived by the Radboud University Medical Centre Ethics Committee. In this study we evaluated all patients who underwent CM for various indications in our center between January 2011 and April 2016. To accurately investigate the rate of voice changes related only to CM and not due to other reasons or pathology, the following patient were excluded from analysis: patients who already had preoperative voice change, patients with voice change only after subsequent pulmonary resection (but not after CM), and patients who underwent CM after induction therapy (Fig. 1). Chart review (medical and nursing records) was performed retrieving data on preoperative characteristics including demographics and oncological details (if appropriate), intraoperative records regarding harvested lymph node (LN) stations during CM, and postoperative data including voice changes and clinical follow-up. Voice changes with full recovery within 14 days were attributed to intubation-related causes [6]. Any otolaryngology (ENT) consultations for voice changes were noted and evaluated, including laryngoscopy performed and voice therapy modalities prescribed.

**Fig. 2:**
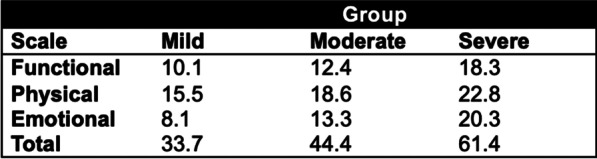
Cut-off scores for categorization of voice impairment severity for the Voice Handicap Index (VHI) [7]

Informed consent forms and follow-up questionnaires were sent to patients with documented voice changes after CM. This was done at least one year postoperatively, evaluating the status of postoperative voice change and its impact on daily living. All participants provided written informed consent. A self-made follow-up questionnaire was constructed (Appendix) to confirm voice changes after CM, assessing possible recovery and recovery period, and appraising maximal hindrance patients experienced from these voice complaints (on a numeric rating scale [NRS] from 0 to 10, 0 depicting no hindrance from the voice changes and 10 representing the most hindrance imaginable). In addition, the standardized 30-item self-administered Voice Handicap Index (VHI) questionnaire was used to assess the impact of voice impairment on a patient's QoL [7, 8]. A 4-point interval score from ‘never’ (0 points) to ‘always’ (4 points) is used to indicate the frequency of various voice complaints. These items can be combined into a total score, and they can be counted separately into three different domain subscores (functional, emotional and physical), with higher scores indicating more severe voice impairment. In addition, cut-off points have been proposed (Fig. 2) to categorize the various scores into either mild, moderate or severe voice impairment [7]. Again, patients were asked to score the items of the VHI questionnaire according to the maximal hindrance patients experienced from these voice complaints. A Dutch version of the questionnaire was distributed by mail early 2018. Initial non-responders were sent the questionnaire again or were contacted by telephone.

### CM

CM was performed according to the international guidelines [9]. After skin incision and dissection of overlying layers, the pretracheal fascia was incised to reach the level of the trachea. Blunt digital dissection was performed to allow entrance into the superior mediastinum, developing a tract between the anterior trachea and posterior to the great vessels. A mediastinoscope was introduced and biopsies were performed of at least three mediastinal nodal stations. VAMLA and TEMLA techniques were not used in this study period. The procedure was performed by three cardiothoracic surgeons (WL, SvdH, AV) with similar standardized CM technique.

### Statistical analysis

Continuous variables are reported as mean ± standard deviation (S.D.), or as median and range. Categorical variables are reported as numbers and percentages. Chi squared and Fisher’s exact test were used for comparing categorical variables between two groups. For comparison of continuous variables between two different groups, an independent Student’s *T*-test was used. In case of non-normal distribution, a Mann–Whitney’s *U* test or Wilcoxon non-parametric test was used for independent samples or related samples respectively.

Multivariable, binary logistic regression models were constructed to determine factors independently associated to predicting factors for CM-related voice changes. Odds ratio (OR) for univariable analysis and adjusted OR for multivariable analysis were calculated to express the contribution of various explanatory variables.

Statistical analysis was performed using Statistical Package for Social Sciences for Windows, version 22.0 (SPSS Inc., Chicago, Ill., USA). A *P*-value < 0.05 was considered statistically significant.

## Results

During the study period, CM was performed in 337 patients. Of these, 67 were excluded due to pre-existing voice changes, and/or underwent neoadjuvant chemo(radio)therapy, and/or had voice changes after pulmonary resection (but not after CM) (Fig. 1). The mean period between CM and pulmonary resection in the total group (before exclusion) was 8.8 ± 11.2 days. The remaining 270 patients were included for final analysis (Table 1). Of these, 65% were male with a mean age of 62.2 years at the time of CM. The majority of patients underwent CM for staging of (suspected) lung cancer (84%). Of the patients with (suspected) lung cancer, 53% had a right-sided tumor, and 42% concerned squamous cell carcinoma.

**Table 1:** Patient characteristics

	Total group (N = 270)	Voice changes after CM lasting > 14 days (N = 17)	No voice changes after CM or full recovery ≤ 14 days (N = 253)	*P*-value
Age, years (during CM, mean ± SD)	62 ± 13	60 ± 15	62 ± 12	0.36
*Gender*				
Female	94 (35%)	6 (35%)	88 (35%)	0.97
*Indication*				0.63
Staging for (suspected) cancer	227 (84%)	15 (88%)	212 (84%)	
Mediastinal lymphadenopathy/mass	43 (16%)	2 (12%)	41 (16%)	
*Location of (suspected) cancer*				0.10
Left	98 (43%)	6 (40%)	92 (43%)	
Right	121 (53%)	7 (47%)	114 (54%)	
Bilateral	8 (4%)	2 (13%)	6 (3%)	
*Stage of cancer*				0.72
I	29 (13%)	3 (20%)	26 (12%)	
II	50 (22%)	3 (20%)	47 (22%)	
III	124 (55%)	7 (47%)	117 (55%)	
IV	17 (8%)	2 (13%)	15 (7%)	
Unknown	7 (3%)	0 (0%)	7 (3%)	
*Histopathology*				< 0.001
Small cell lung cancer	1 (0%)	1 (7%)	0 (0%)	
Adenocarcinoma	86 (38%)	8 (53%)	78 (37%)	
Squamous cell carcinoma	96 (42%)	1 (7%)	95 (45%)	
Large cell lung carcinoma	6 (3%)	0 (0%)	6 (3%)	
Other	38 (17%)	5 (33%)	33 (16%)	
Amount of harvested LNs (mean)	4 ± 1	4 ± 1	4 ± 1	0.48
*Harvested LN stations*	268 (99%)	17 (100%)	251 (99%)	0.71
*Left sided*	237 (88%)	16 (94%)	221 (87%)	0.70
2L	71 (26%)	5 (29%)	66 (26%)	0.77
4L	231 (86%)	16 (94%)	215 (85%)	0.14
*Right sided*	257 (95%)	16 (94%)	241 (95%)	1.00
2R	190 (70%)	12 (71%)	178 (70%)	0.79
4R	255 (94%)	16 (94%)	239 (95%)	0.63
Subcarinal (7)	241 (89%)	16 (94%)	225 (89%)	0.25

Data are shown as mean ± standard deviation (SD), number (percentage) or as reported

*CM* cervical mediastinoscopy, *LN* lymph node

After chart review, 19 patients were identified who experienced voice changes after CM. Of these, two made full recovery within fourteen days and were therefore attributed to intubation-related causes [6]. Of the remaining 17 patients (6.3% of the total group), 16 (94%) complained of hoarseness and one reported dysphonia.

### QoL data and VHI-questionnaires

Follow-up questionnaires were sent to all patients who had experienced voice changes lasting more than 2 weeks after CM. A total of five patients were deceased at the time of the study with a mean period between CM and death of 15.1 ± 8.1 months. Of these patients, two still experienced voice changes at the last documented occasion of follow-up (at 1 and 8 months after CM).

Of the 12 patients who were alive at the time of sending the questionnaires, one was lost to follow-up, 10 completed the self-made follow-up questionnaire and VHI questionnaire (response rate 91%), with a median follow-up time of 33.2 months (range 23.0–83.0 months). Totaling all data from chart review and follow-up through the questionnaires, of the 17 patients who have reported voice changes after CM lasting at least two weeks, seven experienced documented full recovery of their voice change, with a mean time to full recovery of 7.4 ± 3.4 months (median 6.0 months, range 4.2–12.1 months) (Fig. 1). However, at the time of completing the questionnaires, four patients (4/270 = 1.5%) still experienced persistent voice changes more than two years after CM (median follow-up 41.0 months, range 33.2–83.0 months).

The documented maximum hindrance patients experienced from voice complaints during the whole follow-up period was 6.3 ± 3.0 out of 10. Patients with full recovery reported a higher maximum hindrance during follow-up than patients with persistent voice complaints, although not statistically significant (7.5 vs. 4.8, *P* = 0.194).

Results from the VHI questionnaires are stated in Table 2. The mean total VHI score was 43.0 ± 28.7, corresponding with mild to moderate voice impairment severity (Fig. 2). When comparing the scores between patients with persistent voice complaints versus patients with full recovery, patients with full recovery again reported higher scores indicating more severe voice impairment and effect on QoL in the period of their symptoms (55.5 vs. 26.3, *P* = 0.140), and was significantly higher in the functional domain (21.8 vs. 6.5, *P* = 0.016).

**Table 2:** Voice Handicap Index (VHI) and follow-up questionnaire data

	Total group (N = 10)	Full recovery during follow-up (N = 6)	Persistent voice changes (N = 4)	*P*-value
Maximal hindrance patients experienced from voice complaints during follow-up*, mean	6.3 ± 3.0	7.5 ± 3.1	4.8 ± 2.9	0.19
*VHI questionnaire*				
*Total score*	43.8 ± 30.1	55.5 ± 31.9	26.3 ± 18.6	0.14
Functional score	15.7 ± 10.8	21.8 ± 9.2	6.5 ± 4.7	0.02
Physical score	18.8 ± 10.9	21.7 ± 11.9	14.5 ± 8.7	0.34
Emotional score	9.3 ± 10.2	12.0 ± 12.1	5.3 ± 5.7	0.33

Data are shown as mean ± standard deviation (S.D.)

*It is represented on a 0–10 scale, with 0 being least hindrance and 10 most hindrance

### ENT consultations

Twelve of the 17 patients (71%) with postoperative voice changes lasting at least 2 weeks were referred for ENT-consultations. Of these 12 patients, laryngoscopy was performed in 11 (92%), with a mean period of 63.3 ± 58.3 days between CM and laryngoscopy. In all but one case, a paresis of the left vocal cord was documented, resulting in 3.7% confirmed RLN injury for the total study group. In these 10 patients with confirmed RLN injury, bilateral lymph node biopsies were performed in all cases (station 7 100%, station 4R 100%, station 4L 100%, station 2R 80%, station 2L 50%).

**Fig. 3:**
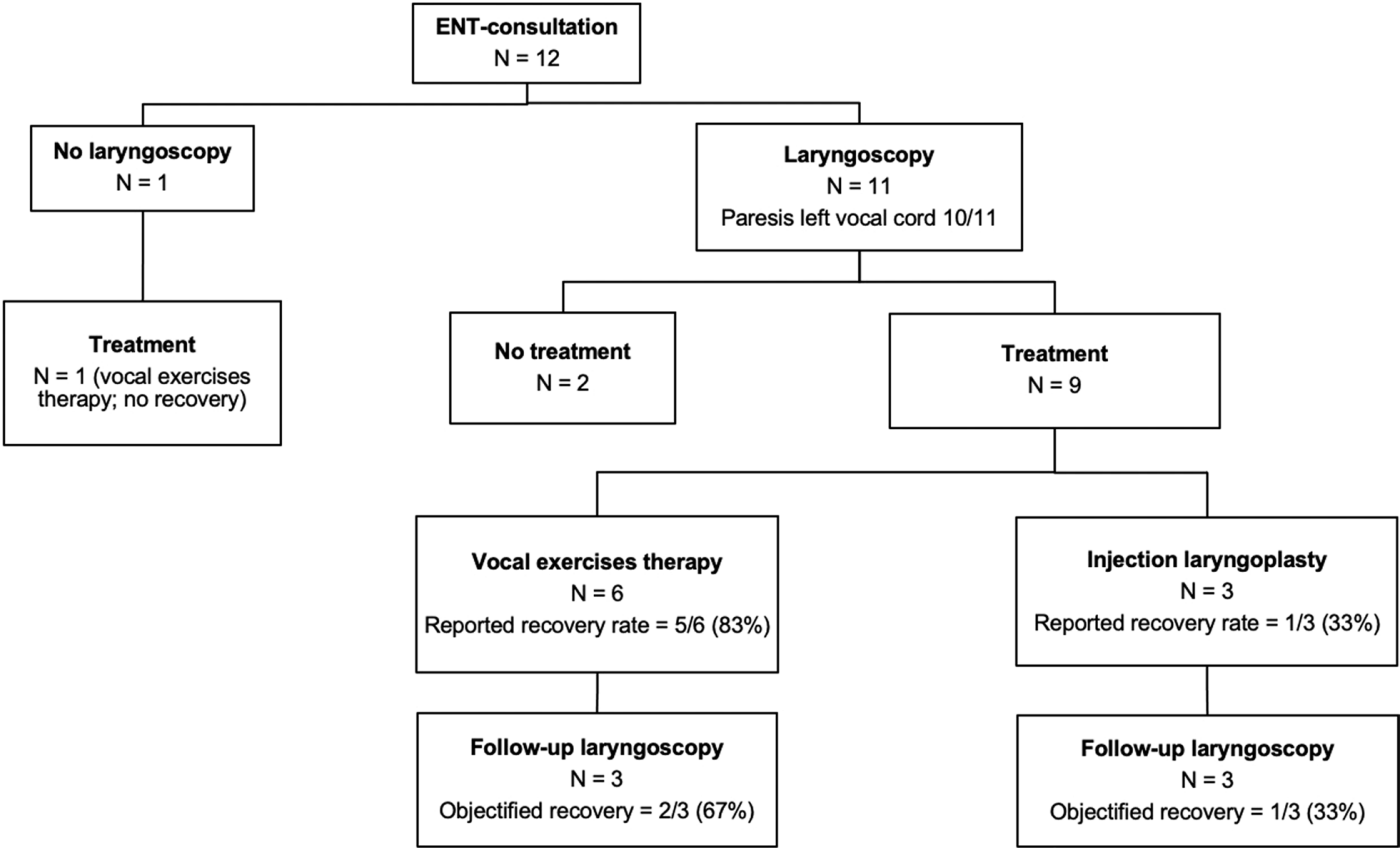
Flow diagram of patients with ENT-consultations. ENT = Otolaryngology

In 10 of the patients referred for ENT-consultations some form of therapy was initiated (83%), including injection laryngoplasty (3/10, 30%) and vocal exercises therapy (7/10, 70%). Vocal exercises therapy provided a recovery rate of 71% with a mean recovery period of 8.6 ± 3.3 months, and injection laryngoplasty had a recovery rate of 33% with a recovery period of 4.6 months. Follow-up laryngoscopy was performed in six patients, showing recovery of the left vocal cord in three patients (Fig. 3). Four of the 7 patients with reported recovery of voice changes underwent a follow-up laryngoscopy, showing recovery of the left vocal cord in 2 patients.

### Predicting persistent postoperative voice changes

In the multivariate analysis exploring risk factors (i.e. age, gender, indication for CM, location of known/suspected cancer, stage of cancer, amount of harvested lymph nodes and harvested lymph node stations) for postoperative voice changes after CM, no significant factors could be identified predicting either the occurrence of voice changes postoperatively, or persistent voice changes more than two years after CM.

## Discussion

In this retrospective cohort study of 270 patients undergoing CM, we found that at least 6.3% of patients experienced voice changes postoperatively which lasted more than 2 weeks, with documented RLN injury in 91% of patients who underwent laryngoscopy. Nevertheless, in four patients (1.5% of the total group), voice complaints persisted for more than two years after surgery. These voice changes led to mild to moderate disabilities in daily living.

The 6.3% incidence of voice changes and 3.7% incidence of confirmed RLN injury after CM in our study is greater than described in the known literature, where RLN injury rates of 0–3% have been reported after CM [3, 10], and even < 1% in larger case series [2, 3]. However, the incidence is dependent on the exact definition of the outcome measure and the intensity of the search for this complication. When routine indirect laryngoscopy was performed after CM in a historical series, Widström found a 6% rate of vocal cord palsy [11], already suggesting this problem might be overlooked during regular clinical follow-up in other reports (although the surgical instrumentation may have been different then). Furthermore, our current study is focused on functional patient-reported outcome, with reviewing of both medical and nursing records, which might be a more accurate method to evaluate the magnitude of this problem. The majority of available reports on RLN injury or palsy after CM [2, 3, 10] rarely provide well-defined data definitions on morbidity outcomes, and it is unclear whether these incidence rates were scored based on functional complaints or laryngoscopy findings, or when the follow-up evaluation was performed.

Multivariable analysis did not reveal any risk factors predicting the occurrence of voice changes after CM, whether temporary or persistent. However, considering the small number of patients, this should be interpreted with caution. Due to the different anatomical course of the left and right RLN, it is mainly the left RLN that is prone for direct injury [12]. Therefore, it is reasonable to assume that left-sided tumors, necessitating a more thorough evaluation of the left paratracheal nodal stations, would pose an increased risk of RLN injury. However, electromyographic monitoring during CM have demonstrated that the most important RLN stimulation occurred during digital blunt dissection of the anterior wall of the trachea, even greater than during direct stimulation with electrocautery during node harvesting [1, 12]. Knowing this mechanism, digital blunt dissection or the introduction of the mediastinoscope should be performed with caution in the hope of reducing RLN injury rates caused by indirect stretch-induced injuries during CM [12].

In the 17 patients with reported voice changes after CM, 71% underwent ENT consultation, and 92% of those underwent laryngoscopy. Left vocal cord palsy was confirmed in all but one of these investigations. Most of the patients who underwent ENT consultations received some form of voice therapy. Which interventions could be effective for voice changes and RLN injury after CM is unclear. However, the setting of RLN injury after thyroid surgery could provide some insight regarding this topic. Various treatment modalities have been recommended in this setting, including “reversible interventions” such as vocal exercises therapy and (absorbable) vocal cord injection augmentation [13, 14], and more aggressive (irreversible) procedures like surgical laryngoplasty procedures for vocal cord palsy persisting more than 1 year [13, 14]. In our study, vocal exercises therapy was most utilized with a recovery rate of 71%.

As mentioned earlier, it has been reported that most RLN injuries during CM are caused by indirect stretch-induced trauma [1, 12]. Therefore, it is reasonable to assume that at least a part of the patients with voice changes are due to RLN neuropraxia rather than complete transection of the nerve, and some recovery can be expected. However, a main finding of our study is that at least 1.5% of our study group exhibited prolonged voice changes after CM due to RLN injury, lasting for more than two years after surgery, despite therapeutic intervention whenever possible. This led to mild to moderate voice handicap affecting QoL and daily living.

Several limitations impact the findings of our study. To the best of our knowledge, this study is the first to present follow-up data on voice changes after CM. However, with the retrospective study setup of our current analysis, it is difficult to report on an exact recovery rate of these voice changes due to the patients lost to follow-up. Of the patients available for follow-up, 63% (7 out of 11) had confirmed full recovery of the voice complaints, all within 12 months after CM. Unfortunately, no other follow-up data is available for comparison in the setting of CM. Additionally, standardized postoperative voice assessments were not performed in our study; although both medical and nursing records were reviewed, the retrospective methodology possibly led to an underestimation of the rate of this complication.

Traditionally, CM remains the diagnostic test with the highest negative predictive value to rule out mediastinal lymph node (N2) disease [9, 15]. In the current guidelines on mediastinal staging for lung cancer patients, confirmatory CM is still indicated in situations of high clinical suspicion of mediastinal metastases if endoscopic staging procedures such as endobronchial (EBUS) and esophageal endosonography (EUS) are negative [9, 15], and is advised to prevent unforeseen N2 disease at surgical resection. However, with the advancements and increasing accuracy of these endoscopic staging techniques, the role of CM has been questioned [10, 16]. In the Netherlands, guideline adherence for performing mediastinoscopy in patients with NSCLC is only 51%, with wide variation between various centers (0–100%) [17]. Until more randomized data will be presented to more exactly elucidate the role of CM in the mediastinal staging algorithms of patients with lung cancer [16], the clinician should educate the patient about the risks of permanent voice changes after CM and its impact on daily living and QoL. Meanwhile, with diminishing numbers of mediastinoscopy procedures performed in various centers, and consequently declining experience per surgeon, attention for potential complications such as RLN injury becomes even more urgent. Furthermore, standardized voice assessments might be advisable considering the underreporting of this complication in the known literature. Patients with postoperative voice changes and suspicion for RLN injury should be referred to ENT specialists for evaluation and consideration of voice rehabilitation strategies.

Areas of interests for future research regarding this issue include preventive measures intraoperatively and the optimal management for established RLN injury after CM. Again, no data is available for these topics specifically for the setting after CM. Regarding management for confirmed RLN injury after CM, there is a lack of evidence for the optimal voice therapy modality. However, our current study did not find any case of recovery of voice changes persisting after one year after surgery, justifying some form of definitive voice treatment after this period (similar to the setting of voice changes after thyroid surgery) [13, 14].

## Conclusions

In conclusion, despite the beforementioned limitations, this study provides the first available follow-up data reporting the risks of voice changes associated with CM, being persistent in 6.3% and permanent in at least 1.5% of patients, leading to mild to moderate disabilities in daily living. Further prospective studies with standardized postoperative voice evaluations and follow-up programs are warranted to clarify the exact magnitude of, as well as preventive measures for this usually underestimated complication.

## Data Availability

The datasets used and/or analyzed during the current study are available from the corresponding author on reasonable request.

## Abbreviations

CM: Cervical mediastinoscopy
RLN: Recurrent laryngeal nerve
TEMLA: Transcervical extended mediastinal lymphadenectomy
VAMLA: Video-assisted mediastinoscopy lymphadenectomy
QoL: Quality of life
LN: Lymph node
ENT: Ear nose throat/Otolaryngology
NRS: Numeric rating scale
VHI: Voice handicap index
S.D.: Standard deviation
OR: Odds ratio
SPSS: Statistical package for social sciences
EBUS: Endobronchial ultrasound
EUS: Esophageal ultrasound
IONM: Intraoperative nerve monitoring

## References

[1] Roberts JR, Wadsworth J (2007). Recurrent laryngeal nerve monitoring during mediastinoscopy: predictors of injury. Ann Thorac Surg.

[2] Wei B, Bryant AS, Minnich DJ, Cerfolio RJ (2014). The safety and efficacy of mediastinoscopy when performed by general thoracic surgeons. Ann Thorac Surg.

[3] Zakkar M, Tan C, Hunt I (2012). Is video mediastinoscopy a safer and more effective procedure than conventional mediastinoscopy?. Interact Cardiovasc Thorac Surg.

[4] Yendamuri S, Demmy TL (2012). Is VAMLA/TEMLA the new standard of preresection staging of non-small cell lung cancer?. J Thorac Cardiovasc Surg.

[5] Yoo DG, Kim YH, Kim DK, Kim HR, Park SI (2011). Clinical feasibility and surgical benefits of video-assisted mediastinoscopic lymphadenectomy in the treatment of resectable lung cancer. Eur J Cardiothorac Surg.

[6] Mendels EJ, Brunings JW, Hamaekers AE, Stokroos RJ, Kremer B, Baijens LW (2012). Adverse laryngeal effects following short-term general anesthesia: a systematic review. Arch Otolaryngol Head Neck Surg.

[7] Jacobson BH, Johnson A, Grywalski C, Silbergleit A, Jacobson G, Benninger MS (1997). The voice handicap inventory (VHI): development and validation. Am J Speech Lang Path.

[8] Carding PN, Wilson JA, MacKenzie K, Deary IJ (2009). Measuring voice outcomes: state of the science review. J Laryngol Otol.

[9] De Leyn P, Dooms C, Kuzdzal J, Lardinois D, Passlick B, Rami-Porta R (2014). Revised ESTS guidelines for preoperative mediastinal lymph node staging for non-small-cell lung cancer. Eur J Cardiothorac Surg.

[10] Bousema JE, van Dorp M, Noyez VJJM, Dijkgraaf MGW, Annema JT, van den Broek FJC (2019). Unforeseen N2 disease after negative endosonography findings with or without confirmatory mediastinoscopy in resectable non-small cell lung cancer: a systematic review and meta-analysis. J Thorac Oncol.

[11] Widström A (1975). Palsy of the recurrent nerve following mediastinoscopy. Chest.

[12] Benouaich V, Marcheix B, Carfagna L, Brouchet L, Guitard J (2009). Anatomical bases of left recurrent nerve lesions during mediastinoscopy. Surg Radiol Anat.

[13] Chen X, Wan P, Yu Y, Li M, Xu Y, Huang P (2014). Types and timing of therapy for vocal fold paresis/paralysis after thyroidectomy: a systematic review and meta-analysis. J Voice.

[14] Lynch J, Parameswaran R (2017). Management of unilateral recurrent laryngeal nerve injury after thyroid surgery: a review. Head Neck.

[15] Postmus PE, Kerr KM, Oudkerk M, Senan S, Waller DA, Vansteenkiste J (2017). ESMO guidelines committee. Early and locally advanced non-small-cell lung cancer (NSCLC): ESMO clinical practice guidelines for diagnosis, treatment and follow-up. Ann Oncol.

[16] Bousema JE, Dijkgraaf MGW, Papen-Botterhuis NE, Schreurs HW, Maessen JG, van der Heijden EH (2018). MEDIASTrial study group. MEDIASTinal staging of non-small cell lung cancer by endobronchial and endoscopic ultrasonography with or without additional surgical mediastinoscopy (MEDIASTrial): study protocol of a multicenter randomised controlled trial. BMC Surg.

[17] Hoeijmakers F, Heineman DJ, Beck N, Klamer J, Tollenaar RAEM, Wouters MWJM (2019). Mediastinoscopy for staging of non-small cell lung cancer: surgical performance in The Netherlands. Ann Thorac Surg.

