# Correction: Long‑term follow‑up of voice changes after cervical mediastinoscopy

**DOI:** 10.1186/s13019-022-01972-x

**Published:** 2022-08-29

**Authors:** Ikram Achbar, Wilson W. L. Li, Simone T. Timman, Stefan M. van der Heide, Olga C. J. Schuurbiers, Erik H. F. M. van der Heijden, Ad F. T. M. Verhagen

**Affiliations:** 1 Department of Cardiothoracic Surgery, Radboud University Medical Centre, P.O. Box 9101, 6500 HB Nijmegen, The Netherlands; 2 Department of Pulmonary Diseases, Radboud University Medical Centre, P.O. Box 9101, 6500 HB Nijmegen, The Netherlands

## Correction to: Journal of Cardiothoracic Surgery (2022) 17:161 https://doi.org/10.1186/s13019-022-01884-w

Following publication of the original article [1], the reference no. 4 has been misplaced in reference no. 11, reference 4 should as “Widström A. Palsy of the recurrent nerve following mediastinoscopy. Chest. 1975;67(3):365–6.” and other references will be sequenced orderly and the in text citiation will be changed as below.

The in text citation for the text has been changed from

Additionally, when the extensiveness of mediastinal lymph node dissection is increased, i.e. with transcervical extended mediastinal lymphadenectomy (TEMLA) or video-assisted mediastinoscopic lymphadenectomy (VAMLA) techniques, the risk of RLN injury also rises [4]. In a contemporary series with 108 patients after VAMLA, recurrent nerve palsy was identified in 5% of patients [5].

to

Additionally, when the extensiveness of mediastinal lymph node dissection is increased, i.e. with transcervical extended mediastinal lymphadenectomy (TEMLA) or video-assisted mediastinoscopic lymphadenectomy (VAMLA) techniques, the risk of RLN injury also rises [5]. In a contemporary series with 108 patients after VAMLA, recurrent nerve palsy was identified in 5% of patients [6].

The in text citation for the text has been changed from

Chart review (medical and nursing records) was performed retrieving data on preoperative characteristics including demographics and oncological details (if appropriate), intraoperative records regarding harvested lymph node (LN) stations during CM, and postoperative data including voice changes and clinical follow-up. Voice changes with full recovery within 14 days were attributed to intubation-related causes [6].

to

Chart review (medical and nursing records) was performed retrieving data on preoperative characteristics including demographics and oncological details (if appropriate), intraoperative records regarding harvested lymph node (LN) stations during CM, and postoperative data including voice changes and clinical follow-up. Voice changes with full recovery within 14 days were attributed to intubation-related causes [7].

The in text citation for the text has been changed from

In addition, the standardized 30-item self-administered Voice Handicap Index (VHI) questionnaire was used to assess the impact of voice impairment on a patient’s QoL [7,8]. A 4-point interval score from ‘never’ (0 points) to ‘always’ (4 points) is used to indicate the frequency of various voice complaints. These items can be combined into a total score, and they can be counted separately into three different domain subscores (functional, emotional and physical), with higher scores indicating more severe voice impairment. In addition, cut-off points have been proposed (Fig. 2) to categorize the various scores into either mild, moderate or severe voice impairment [7].

to.

In addition, the standardized 30-item self-administered Voice Handicap Index (VHI) questionnaire was used to assess the impact of voice impairment on a patient’s QoL [8,9]. A 4-point interval score from ‘never’ (0 points) to ‘always’ (4 points) is used to indicate the frequency of various voice complaints. These items can be combined into a total score, and they can be counted separately into three different domain subscores (functional, emotional and physical), with higher scores indicating more severe voice impairment. In addition, cut-off points have been proposed (Fig. 2) to categorize the various scores into either mild, moderate or severe voice impairment [8].

The in text citation for the text has been changed from

CM was performed according to the international guidelines [9].

to

CM was performed according to the international guidelines [10].

The in text citation for the text has been changed from

After chart review, 19 patients were identified who experienced voice changes after CM. Of these, two made full recovery within fourteen days and were therefore attributed to intubation-related causes [6].

to

After chart review, 19 patients were identified who experienced voice changes after CM. Of these, two made full recovery within fourteen days and were therefore attributed to intubation-related causes [7].

The footnotes for the Fig. 2 has been changed from

Cut-off scores for categorization of voice impairment severity for the Voice Handicap Index (VHI) [7]

to

Cut-off scores for categorization of voice impairment severity for the Voice Handicap Index (VHI) [8]

The in text citation for the text has been changed from

The 6.3% incidence of voice changes and 3.7% incidence of confirmed RLN injury after CM in our study is greater than described in the known literature, where RLN injury rates of 0–3% have been reported after CM [3, 10], and even < 1% in larger case series [2, 3]. However, the incidence is dependent on the exact definition of the outcome measure and the intensity of the search for this complication. When routine indirect laryngoscopy was performed after CM in a historical series, Widström found a 6% rate of vocal cord palsy [11], already suggesting this problem might be overlooked during regular clinical follow-up in other reports (although the surgical instrumentation may have been different then). Furthermore, our current study is focused on functional patient-reported outcome, with reviewing of both medical and nursing records, which might be a more accurate method to evaluate the magnitude of this problem. The majority of available reports on RLN injury or palsy after CM [2, 3, 10] rarely provide well-defined data definitions on morbidity outcomes, and it is unclear whether these incidence rates were scored based on functional complaints or laryngoscopy findings, or when the follow-up evaluation was performed.

to

The 6.3% incidence of voice changes and 3.7% incidence of confirmed RLN injury after CM in our study is greater than described in the known literature, where RLN injury rates of 0–3% have been reported after CM [3, 11], and even < 1% in larger case series [2, 3]. However, the incidence is dependent on the exact definition of the outcome measure and the intensity of the search for this complication. When routine indirect laryngoscopy was performed after CM in a historical series, Widström found a 6% rate of vocal cord palsy [4], already suggesting this problem might be overlooked during regular clinical follow-up in other reports (although the surgical instrumentation may have been different then). Furthermore, our current study is focused on functional patient-reported outcome, with reviewing of both medical and nursing records, which might be a more accurate method to evaluate the magnitude of this problem. The majority of available reports on RLN injury or palsy after CM [2, 3, 11] rarely provide well-defined data definitions on morbidity outcomes, and it is unclear whether these incidence rates were scored based on functional complaints or laryngoscopy findings, or when the follow-up evaluation was performed.

The in text citation for the text has been changed from

Traditionally, CM remains the diagnostic test with thehighest negative predictive value to rule out mediastinallymph node (N2) disease [9, 15]. In the current guidelineson mediastinal staging for lung cancer patients, confirmatoryCM is still indicated in situations of high clinicalsuspicion of mediastinal metastases if endoscopic stagingprocedures such as endobronchial (EBUS) and esophagealendosonography (EUS) are negative [9, 15], and is advised to prevent unforeseen N2 disease at surgical resection. However, with the advancements and increasingaccuracy of these endoscopic staging techniques, the role of CM has been questioned [10, 16].

to

Traditionally, CM remains the diagnostic test with thehighest negative predictive value to rule out mediastinallymph node (N2) disease [10, 15]. In the current guidelineson mediastinal staging for lung cancer patients, confirmatoryCM is still indicated in situations of high clinicalsuspicion of mediastinal metastases if endoscopic stagingprocedures such as endobronchial (EBUS) and esophagealendosonography (EUS) are negative [10, 15], and is advised to prevent unforeseen N2 disease at surgical resection. However, with the advancements and increasingaccuracy of these endoscopic staging techniques, the role of CM has been questioned [11, 16].

The original article has been corrected.
